# Precision of provider licensure data for mapping member accessibility to Medicaid managed care provider networks

**DOI:** 10.1186/s12913-018-3776-4

**Published:** 2018-12-17

**Authors:** Nathaniel Bell, Ana Lòpez-DeFede, Rebecca C. Wilkerson, Kathy Mayfield-Smith

**Affiliations:** 1 College of Nursing, University of South Carolina, Columbia, SC 29208 USA; 2 Division of Integrated Health and Policy Research, Institute for Families in Society, University of South Carolina, Columbia, SC 29208 USA

**Keywords:** Medicaid, Nurse practitioners, Geocoding, Licensure, Patient-centered medical homes

## Abstract

**Background:**

In July 2018, the Centers for Medicare and Medicaid Services (CMS) updated its Medicaid Managed Care (MMC) regulations that govern network and access standards for enrollees. There have been few published studies of whether there is accurate geographic information on primary care providers to monitor network adequacy.

**Methods:**

We analyzed a sample of nurse practitioner (NP) and physician address data registered in the state labor, licensing, and regulation (LLR) boards and the National Provider Index (NPI) using employment location data contained in the patient-centered medical home (PCMH) data file. Our main outcome measures were address discordance (%) at the clinic-level, city, ZIP code, and county spatial extent and the distance, in miles, between employment location and the LLR/NPI address on file.

**Results:**

Based on LLR records, address information provided by NPs corresponded to their place of employment in 5% of all cases. NP address information registered in the NPI corresponded to their place of employment in 64% of all cases. Among physicians, the address information provided in the LLR and NPI corresponded to the place of employment in 64 and 72% of all instances. For NPs, the average distance between the PCMH and the LLR address was 21.5 miles. Using the NPI, the distance decreased to 7.4 miles. For physicians, the average distance between the PCMH and the LLR and NPI addresses was 7.2 and 4.3 miles.

**Conclusions:**

Publicly available data to forecast state-wide distributions of the NP workforce for MMC members may not be reliable if done using state licensure board data. Meaningful improvements to correspond with MMC policy changes require collecting and releasing information on place of employment.

## Background

In July 2018, the Centers for Medicare and Medicaid Services (CMS) updated its Medicaid Managed Care (MMC) regulations to ensure adequate network and access standards for enrollees for access to primary care as well as a host of specialist, hospital, and pharmacological services [1]. The rule updated the 2016 federal policy underlying the need that states report on the availability and accessibility of services provided through their MMC networks [2]. In total, 11 different types of providers and provisions are regulated. Although the new regulations largely make official requirements that were already a staple of state MMC contract practices, it adds greater regulatory oversight and accountability to how states design their managed care and utilize contractors [3].

Many see MMC network adequacy standards as a means to help beneficiaries more easily navigate and use their coverage [4–6]. In particular, Medicaid recipients have consistently reported less timely access to health care services than other population groups [7–9]. They are also a population group who more often require treatment for complex health conditions, many of which go untreated due to barriers to care access [10, 11]. CMS emphasizes that such standards will help to protect the long-term health outcomes among beneficiaries by making it more possible to ensure better access to primary and preventive care services [12].

Although many different thresholds underline CMS requirements to ensure adequate access to care, enrollee distance and drive time standards to providers are the most common feature of state contracts [13]. MMC travel and distance standards are allowed to vary by state as well as by provider and service specialty. Maximum distance standards to primary care physicians range from 5 miles in metropolitan areas throughout Arizona to within 60 miles among Frontier areas in New Mexico [14]. Similar thresholds are in place for travel times. For some states, plans are required to demonstrate that a majority of members (e.g., 90%) can access network providers within specific thresholds. The updated federal rule does not change the travel time or distance parameters previously established by the state.

One challenge of monitoring current travel time and distance standard calculations is the evolving care team complexity. Historically, states have monitored care capacity through calculating provider-to-population ratios of current providers to expected enrollees, or through applying geo-mapping algorithms to calculate distance from enrollees to providers in miles and minutes of drive time. Although geo-mapping algorithms make it feasible to estimate the time and distance MMC populations must travel to obtain healthcare services, MMCs contract with many different types of providers. In particular, Medicaid programs are increasingly relying on nurse practitioners (NPs) for primary care delivery [15–17], particularly for rural and vulnerable populations [18, 19]. This increase is in large part a response to mounting pressures on primary care delivery, particularly in communities with provider shortages.

At issue is that the accuracy of information on NP practice locations is not easily verifiable. For example, many states currently exclude NPs from workforce assessments and forecast projections because they are not universally considered autonomous primary care providers [20]. The same rationale excludes NPs from federally defined health professional shortage areas (HPSA) calculations [21] despite consistently outpacing physicians in improving primary care capacity in these areas [22]. An additional challenge stems from the structure of NP licensure data itself, which often does not differentiate whether the registry address reflects where NPs practice or where they reside. For example, in one of the most comprehensive analysis of NP practice distribution to date, the authors could not determine whether the licensure data from 11 of 12 state workload assessments represented where NPs practiced [23].

Other licensure data sources may also be limited. For instance, all clinicians who elect to participate with CMS are required to have a National Provider Identifier (NPI). However, CMS does not require a clinician to use their personal or professional address when registering their NPI, and no flag is provided that describes the address type provided. Nor is there any requirement that a provider needs to update their address information after switching places of employment. The potential impact of this limitation may not be trivial. One study investigating positional error in address information listed in the national physician masterfile found that nearly 40% of the mailing addresses were over 6 miles from their corresponding practice location [24].

These challenges aside, a key benefit of licensure data to Managed Care Organizations (MCO) is that they are publicly available, population based, and are released with geographic identifiers. As multiple MCOs participate in state MMC delivery, access to state-wide data on the entire NP workforce allows individual MCOs to actively target network expansion areas based on NP workforce locations. At the same time, NPs are a limited resource for many states owing to migration toward states with fewer practice regulations. Statewide data sources that represent NP workforce distributions therefore could come to play a potentially pivotal role for advancing alternative MMC models for primary care delivery.

Another benefit of state licensure data is that it is the only source of information available to link NPs to their precepting/supervising physicians. Twenty states have collaborative practice agreements that require NPs to work under physician supervision [25, 26]. Of these, eight require extended supervision for a period of time (e.g., 2 years), and five limit the number of NPs a physician can supervise (e.g., 2 NPs). At least four of these states also require NPs to practice within the same clinic or specific geographic distance of the physician, thus potentially restraining provider penetration into shortage areas. Each of these benefits, however, requires access to accurate information on nursing workforce distributions.

The reliance on administrative data to forecast drive times and distances to providers, coupled with growing complexity in care teams who participate in MMCs, underscores the need to refocus attention on available data for monitoring the spatial distribution of the NP workforce. Of particular relevance is whether publicly available state licensure board data accurately represent the distribution of the workforce. To our knowledge, little comparable research exists as to whether licensure address information registered by NPs corresponds with where they work or where they live. As such, the objective of this study was to evaluate the frequency in which geocoded licensure data for the NP workforce corresponded to their place of employment. To estimate the reliability of licensure data for representing provider practice locations, we “ground truthed” our analysis using practice location data recorded in the National Committee for Quality Assurance (NCQA) patient-centered medical home (PCMH) provider file. The PCMH administrative file is one of the few publicly available sources of information that can be used to confirm whether the address information obtained by state represents where providers practice. We examined the agreement and discordance (e.g., %) between addresses listed in the PCMH file against two publicly available data sources frequently used to map the NP workforce: state licensure board (LLR) records and the national provider identifier (NPI) file. For comparison purposes, we contrasted our findings against LLR and NPI address information recorded for primary care physicians.

## Methods

### Data sources

Our evaluation is based on 2017 South Carolina (SC) NP and physician workforce data. Each year, SC nurses and physicians are required to register or renew their clinical license in order to qualify as a practitioner in the state they wish to practice. Statutory language as to what constitutes a right to practice differs from jurisdiction to jurisdiction (e.g., primary state of residence, demonstration of continuing education, practice hours, etc.). In SC, the public can request an electronic licensee roster from each licensure board for ten dollars. A limited number of data elements are provided in the LLR roster. These include the clinician’s first, middle, and last name; their state licensure number; credential information (e.g. family practice, certified nurse midwife); whether they are board certified for any of their credentials; as well as street-level address information. The LLR does not specify whether the mailing address provided corresponds with a practice location or a personal mailing address.

The NPI is a numeric identifier assigned to healthcare providers who elect to provide services to individuals covered under CMS. It is a 10-digit permanent number. Each month, CMS provides an updated NPI release file that is downloadable through the National Plan and Provider Enumeration System (NPPES). The NPI contains elements such as provider first, middle, and last name; a taxonomy description that specifies their credential type; the date in which the provider’s information was last updated by CMS; entry space for the provider to list their license number, provider, and state in which they practice; and address-level information pertaining to a mailing address. CMS does not require that a clinician use their personal or professional address and no flag is provided that describes the address type provided. The address field represents where the clinician elects to be contacted by CMS regarding changes/information concerning Medicare/Medicaid program.

The PCMH data feed file is distributed by the NCQA. Our research group receives a monthly data feed file from the NCQA containing practice-level identification numbers, the practice name, recognition level (e.g., Level 3), number of clinicians, certification/expiration year, address, as well as provider-level information for employees, including provider name, their NPI, and credential (e.g. MD, NP). These data elements are also publicly available through the NCQA website [27]. We used the September 2017 LLR, NPI, and NCQA files to ensure currency in the data linkages.

### Geocoding

Geocoding, also called address matching, is a widely used methodology to map the geographic distribution of health care providers and for identifying neighborhoods or regions where populations are under-served [28–30]. In this approach, electronic databases containing personal identifiers such as address information are spatially linked to situs (i.e., point), linear (i.e., streets), or area (i.e., Census tracts) boundaries and assigned corresponding latitude and longitude coordinates. The quality of the address data available for mapping health-related events has long been a notable point of research interest within the geographic, computer science, and mathematical disciplines [31]. This has simply not been the case within network adequacy studies. The lack of research would not necessarily be as significant a problem if the address information contained in provider registries only represented places of employment.

Our analysis employed a composite geocoding methodology, which allowed for situs, linear, and area referencing. We linked address information (e.g., street name, street suffix, ZIP code) provided in the LLR, NPI, and PCMH databases to street centerline data using the ESRI commercial geocoding software. For the linkages, we used the ESRI Street Map Premium Address File, which is an enhanced version of commercial street reference data from HERE, TomTom, and INCREMENT P. The benefit of purchasing enhanced centerline files is access to more precise and up-to-date address information. Prior to geocoding, we standardized each address file to US Postal Service mailing format to increase the likelihood of matching the provider address information between files and with the street centerline file. Standardization was done using ZP4 address correction software.

### Data linkages

The address-standardized geocoded data files were linked in SAS using SQL scripting language. Only records that had accurate licensure information across all three registries were included in the analysis. Prior to linking each data file, we used the VLOOKUP function in Microsoft Excel to vet the LLR and NPI licensure numbers listed in the PCMH. We flagged and amended all instances where the PCMH documented the provider’s LLR or NPI incorrectly. As the PCMH file is an administrative file and not used for billing purposes, we presumed the license number contained in the LLR and NPI to be correct. We used provider first, middle, and last names to confirm instances where there was a license mismatch.

We used the SPEDIS procedure in SAS software to identify potential matches that would have been missed due to discrepancies in spelling that may not have been corrected using the ZP4 software (i.e., crossing vs. xing) as well as trailing suffixes (i.e., 100 Main St. vs. 100 Main St. Suite 202B). The SPEDIS function is a form of fuzzy matching; it determines the likelihood of a match between the target characters and returns a score ranging from 0 to 100 [32]. The value 0 signifies a perfect match. We used visual observation of the records to identify a score in which unmatched practice-level addresses were referencing the same location and no false-positive matches were included. We report address matches with and without use of the SPEDIS procedure.

### Regional designation

After geocoding, we categorized each practice as a rural, suburban, or urban PCMH by spatially assigning the practice location to its corresponding US Census Bureau ZIP code tabulated area (ZCTA). ZCTAs approximate US Postal Service ZIP codes and are defined by the Census for statistical purposes. In our evaluation, we created ZCTA-level class breaks in order to maximize spatial correlation with county-level classification system based on Census Metropolitan and Micropolitan Statistical Area definitions as well as to highlight variation within counties. Classifications were based on the percentage of the ZCTA’s total population that was urban as per the Census 2010. Urban Areas were defined as ZCTAs with an urban population comprising more than 72.5% of the total population. Suburban ZCTAs were defined from an urban population comprising between 43.0 and 72.5% of the total population. A ZCTA was designed as a rural area if its urban population percentage was less than 43.0%. All 424 of the state’s ZCTAs were designated. Our classification breakdowns for state ZCTAs was internally determined. The objective was to ensure that ZCTAs accurately corresponded to county classifications, while also representing the rural-urban distribution at a smaller aggregate unit (e.g., urban ZCTAs within a rural county would still be urban, even if surrounded by rural areas).

### Primary study variable

Our primary study variable was an indicator of accuracy between LLR, NPI and PCMH address fields. Accuracy was measured as a discrete variable to estimate overall agreement as well as a continuous variable, in miles, to measure positional error between the PCMH location and the location of the providers mailing address.

### Analysis

Our study was observational. Discrepancies were evaluated using cross tabulations and radar plots. As a provider could be employed at more than one PCMH, we considered an address match to be correct if at least one of the addresses listed in the PCMH file matched the address information in the LLR or NPI. In these instances, we dropped all additional practice locations from the comparisons in order to avoid deflating the denominator used to assess the overall match rates. All analyses were conducted in ArcGIS, version 10.4 and SAS, version 9.4.

## Results

### Overall provider workforce numbers and data linkage matches

In September 2017, a total of 938 physicians and 171 NPs were employed within one or more PCMHs that are recognized as either a family practice/general medicine, internal medicine, or pediatric medical home. This workforce distribution represents approximately 15% of the state’s 6387 physicians with a primary specialty in these fields of medicine and 5% of the state’s 3745 NPs who are actively practicing in the state (e.g., having a documented primary physician supervisor). After linking the LLR linkage to the PCMH file, the number of physician and NP address files with matching licensure information was reduced to 912 and 168 (97 and 98% record retention). After linkage to the NPI file, the number of physician and NP address records with matching LLR, NPI, and PCMH licensure information was reduced to 880 and 162 (94 and 95% record retention). These were the final numbers used for all subsequent evaluations. Prior to all data linkages, we manually edited the licensure numbers recorded in the PCMH file for 248 providers. An additional 17 NPI numbers in the PCMH file were also amended.

### Accuracy of licensure data based on address concordance

Statistics shown in Table 1 correspond to the overall agreement between the LLR, NPI, and PCMH address fields at different geographic scales. For all providers, the match statistics increased in a stepwise fashion as the precision of the analysis decreased (e.g., moving from the clinic location to the county where the PCMH was located). Overall, less than 2% of the LLR records for NPs corresponded to the PCMH where they were employed. In comparison, 44% of the NP addresses listed in the NPI matched the address provided in PCMH file. When evaluated at the county scale, 64% of LLR records corresponded with the county of the PCMH. At the same geographic scale, 86% of the NPI records corresponded with the county of the PCMH.

**Table 1:** Comparison of address concordance among Nurse Practitioner and Physician LLR and NPI files with employment address locations recorded in state PCMH data file

Provider	Scale	Address matches^a^				With SPEDIS address matching^b^			
		LLR (n, %)		NPI (n, %)		LLR (n, %)		NPI (n, %)	
Nurse practitioner									
	Practice	3	1.9	72	44.4	8	4.9	103	63.6
	City	36	22.2	115	71.0	–		–	
	ZIP code	58	35.8	124	76.5	–		–	
	County	104	64.2	140	86.4	–		–	
Physician									
	Practice	506	57.5	416	47.3	627	71.3	632	71.8
	City	700	79.6	702	79.8	–		–	
	ZIP code	752	85.5	776	88.2	–		–	
	County	784	89.1	810	92.0	–		–	

^a^All percentages derived from an N of 880 for physicians and an N of 162 for nurse practitioners

^b^All fuzzy-matches using the SPEDIS procedure in SAS on practice-level comparisons were evaluated using a tolerance score of <= 32

The SPEDIS procedure improved the practice-level matching for all providers. We found that a SPEDIS score value of ‘<=32’ increased the practice-level matches between the registries without resulting in any false-positive matches. The SPEDIS procedure was not used to improve the overall match rate at the city or county scale because of prior data cleaning. It was not used for ZIP code matches because of meaningful differences in numeric values.

Table 2 contrasts address match statistics for NPs and physicians after defining a medical home as an urban, suburban, or rural practice. On average, urban discordance was lower in urban areas for NPs than for either suburban or rural providers. Similar differences existed by licensure data source. For example, LLR data for only one NP provider (3.2%) who practiced in a rural area corresponded to the place of employment, whereas 65% of all NPI addresses for rural NPs corresponded to employment location. Similar trends existed for physician licensure data. With the exception of rural physician practice locations using the NPI, the concordance between PCMH and both licensure files was higher among physicians for all geographic types. All ZCTA comparisons used the SPEDIS match scores for practice level comparisons.

**Table 2:** Comparison of address concordance among Physician and NP LLR and NPI licensure files within urban, suburban, and rural PCMHs

Provider	Data file & scale	ZCTA Classification					
		Urban (n, %)		Suburban (n, %)		Rural (n, %)	
Nurse Practitioner							
	LLR						
	Practice	7	6.9	0	0.0	1	3.2
	City	21	20.8	8	21.6	7	22.6
	ZIP code	42	41.6	10	27.0	7	22.6
	County	73	72.3	16	43.2	18	58.1
	NPI						
	Practice	65	64.4	18	49.6	20	64.5
	City	71	70.3	24	64.9	20	64.5
	ZIP code	80	79.2	25	67.6	20	64.5
	County	91	90.1	28	75.7	22	77.4
Physician							
	LLR						
	Practice	488	70.3	83	66.9	56	57.1
	City	545	78.5	87	70.2	68	69.4
	ZIP code	597	86.0	91	73.4	65	66.3
	County	621	89.5	95	76.6	82	83.7
	NPI						
	Practice	481	69.3	91	73.4	60	61.2
	City	541	78.0	93	75.0	68	69.4
	ZIP code	615	88.6	97	78.2	65	66.3
	County	641	92.4	101	81.5	85	86.7

Physician *N* = 880, Nurse Practitioner *N* = 162

**Fig. 1:**
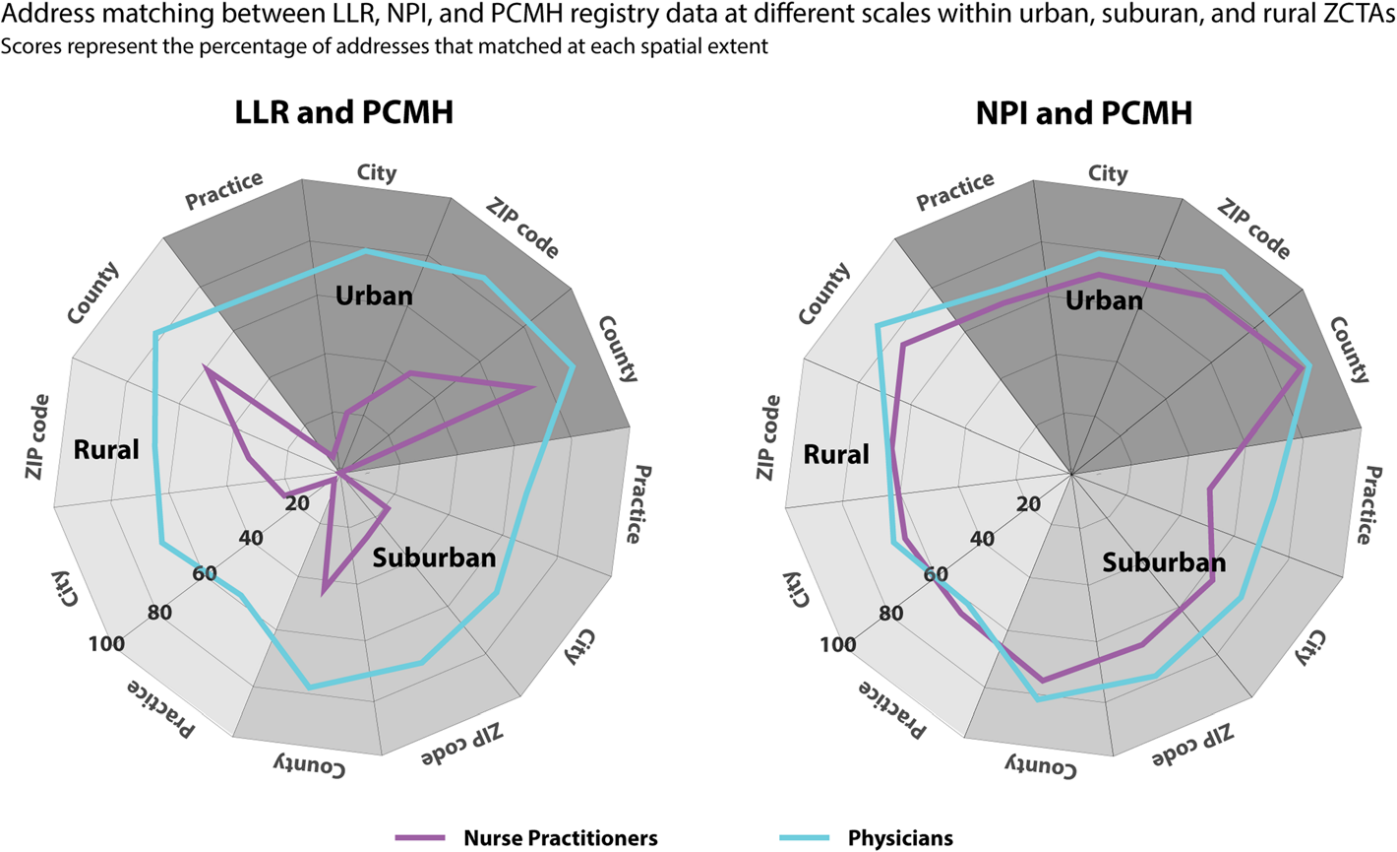
Radar graphs showing the registry address concordance for all providers at the practice-level, city, ZIP code, and county-level spatial extent across urban, suburban, and rural areas. Scores represent the percentage of all matching address files (e.g., 0 = 0% of addresses matched, 100 = 100% of addresses matched)

The data used to build Table 2 is shown graphically in Fig. 1 using a radar graph. For interpretation, greater concentricity (e.g., roundness) in the lines represents more uniform address concordance at each spatial scale and for each area type. The further the data lines are to the outer edge of the graph represents greater address concordance between the address information provided in the LLR/NPI and the employment address of the PCMH.

### Spatial error of licensure data

Spatial error across the registries is shown in Table 3. Spatial error was measured as the network distance, in miles, between the location of the PCMH and the geocoded address registered by the provider in the LLR/NPI. Statistics shown represent the average, standard deviation, minimum, and maximum amount of displacement between both address locations.

**Table 3:** Positional error (in miles) between the address information provided in registries and to the address location of the PCMH. All statistics are stratified by urban, suburban, and rural PCMHs

Data file	Statistic	Nurse Practitioners			Physicians		
		Urban	Suburban	Rural	Urban	Suburban	Rural
LLR							
	Mean spatial error	19.2	25.3	24.7	7.1	7.6	7.5
	SD	57.7	36.5	39.1	43.1	25.2	15.0
	Min. spatial error	0.0	0.7	0.0	0.0	0.0	0.0
	Max. spatial error	538.1	220.3	227.1	735.9	230.4	106.0
NPI							
	Mean spatial error	4.7	8.1	15.9	3.8	4.5	7.9
	SD	13.1	13.8	44.2	11.3	13.1	19.6
	Min. spatial error	0.0	0.0	0.0	0.0	0.0	0.0
	Max. spatial error	118.2	45.1	242.5	188.1	119.5	141.7

For all providers, the positional error depended on whether the address information was derived from the LLR or NPI. Positional error also varied among urban, suburban, and rural practices. In almost every instance, positional error increased when moving from urban to rural locations. For NPs, the greatest amount of positional error in the LLR was among providers in suburban and rural areas, with an average difference of 25.3 and 24.7 miles between the medical home where the provider worked and where they received their license. When derived from the NPI, spatial error decreased to 8.1 and 15.9 miles, respectively. There were few discrepancies in the positional error among physician addresses based on LLR data across urban, suburban, and rural practices, ranging from 7.1 to 7.6 to 7.5 miles, respectively. Based on NPI data, positional error in the physician file decreased to an average of 3.8 and 4.5 miles among urban and suburban providers and increased to 7.9 miles among rural providers.

### Sensitivity analysis of address matching concordance

We conducted a sensitivity analysis to determine whether the results were potentially biased as a result of providers not updating their address information with CMS. We used the NPI “last update” field to assess the whether there was an inverse association between the duration, in days, since a provider last updated their contact information with CMS and the address matching rate. All evaluations were based on SPEDIS-amended data fields.

NPs whose address did not match the address of the PCMH had gone longer since submitting their last update to the NPI (1157 days [970] vs. 1022 days [830]), but these differences were not statistically significant (p 0.330). Physicians whose address did not correspond to the PCMH where they worked had updated their profile more recently than those whose address did match the PCMH, but these differences were not statistically significant (1287 days [1007] vs. 1315 days [1014]; p 0.682).

## Discussion

Key network adequacy requirements specified in the 2016 federal regulations for MMC took effect in 2018. Although most states have historically held MCOs to minimum access and accountability standards, the recent federal rule underlies the need to ensure adequate access and accessibility for providers who elect to participate in MMC. The recent federal rule maintains that states continue to evaluate and report access statistics and geographic relationships between enrollees and their healthcare providers. Despite widespread adoption of geographic accessibility standards, and a federal mandate to amend accessibility regulations, to our knowledge, this study is the first study to evaluate the accuracy of publicly available data for monitoring the geographic distribution of available primary care providers who could participate in MMC.

We found little evidence that state licensure data accurately reflects the workforce location of NPs. This limitation is significant given the importance of licensure data for mapping the location of primary care providers, assessing geographic relationships between NPs and physician preceptors, as well as forecasting gaps in network adequacy. At issue is that the lack of contextual information as to whether the address information in the LLR corresponds to a personal mailing address or a place of employment. This limitation decreases the accuracy of any attempt to estimate where providers actually practice. As shown in this analysis, the potential for incorrect assignment of the NP workforce distribution based on state licensure data is substantial.

Our evaluation suggests that state licensure data may be a poor source of information for forecasting the location of the entire NP workforce. We found that less than 5 % of all addresses in the LLR corresponded to an NPs place of employment. Even at the county level, nearly 40% of the addresses provided to the LLR do not correspond to the county where NPs practiced. By comparison, the NPI data are more accurate. The 12-fold increase in practice-level matches for NPs based on the address data registered with CMS suggests that these data are better suited for forecasting workforce needs. However, even after address cleaning and standardization we could only obtain practice-level matches for 64% of all NPs using the NPI. This level of accuracy raises concerns over the ability to conduct high-level or granular spatial analyses from the available data.

The potential significance of the poor spatial accuracy may be greatest for monitoring network adequacy needs for rural populations. Our analysis showed a 1.3-fold decrease in accuracy for rural NP LLR addresses and over a 3-fold decrease in accuracy for the same providers based on NPI data. In effect, this distinction could potentially create under estimations of care needs if based on provider-to-population ratios, particularly if urban providers are artificially being shifted into a rural county simply due to poor address information. Moving forward, systems that choose to use provider-to-enrollee standards for forecasting care needs will need to consider that such practices could under or over inflate capacity estimates.

Accounting for the geographic detail used in this analysis necessitates a substantial amount of data cleaning and standardization. Our manual inspection of the data found a number of errors in the licensure data entered into the NCQA data file compared to the registries. In addition, the rate of practice-level address agreement improved substantially through using SPEDIS fuzzy-matching techniques without generating any false positive matches. The result of these processes warrants consideration as a standard methodological approach for linking disparate data files for mapping workforce distributions.

These discrepancies may also hold some hidden benefits. Although we primarily interpret from this analysis that the LLR is a very poor information source for mapping the NP workforce distribution, another interpretation is that it is potentially a very good source of information for mapping the potential workforce distribution. If the LLR is in fact representing the place of residence of the NP workforce then these data could potentially be more useful for looking at the workforce capacity in those areas with respect to scope of practice limitations. The subtlety within this context is important given that scope of practice regulations on NPs practicing in SC are some of the most stringent in the country [25].

Based on these findings, the data suggest that NP workforce projections and distribution estimates are most reflective of the actual workforce location when based on county-level data. One opportunity to improve the granularity of the available data would be to advocate for nursing licensure boards to begin recording place of employment information among its members as well as making this information publicly available. From the available literature, this appears to be the practice in the state of Texas, but we are unaware if any other state has taken a similar approach [23]. To some extent, databases such as the NPI as well as the Physician Masterfile avoid this problem by asking clinicians to record whether the registered mailing address represents the location of a clinical practice. This helps, but there is no mandate that the workplace address must be entered and no flag is distributed in the NPI that specifies whether an employment or residential address was provided. Nor is there any mandate with the NPI that a provider needs to update their address information after switching places of employment. At the same time, NPs themselves may be the best advocates for amending licensure board data requirements, particularly if the lack of information provided in the registry artificially excludes them from network adequacy forecasts or workforce expansions.

### Limitations

The findings from this study do come with four important limitations. First, our findings may be generalizable to the workforce that specializes in family practice, internal medicine, and pediatrics. However, there is no a priori reason to presume that the accuracy of licensure or identification files varies according to a provider’s chosen specialty. Additional evaluations based on Medicaid claims data could be used to further distinguish whether these patterns are similar among a larger provider sample. Second, because we could not determine whether each provider was either employed as a full- or part-time clinician we could not estimate whether poor address concordance was due to employment elsewhere or in multiple sites. This is one potential explanation for the poor address match rate based on LLR data. However, the 12-fold increase in address matching among the same provider within the NPI reduces the likelihood that this bias affected our analysis given that a provider can only register a single NPI with CMS. A third and related limitation stems from the use of the PCMH address file to verify provider employment locations. Approximately 15% of all physicians and 5% of all NPs practice within a recognized PCMH. The transition to PCMHs is a relatively recent phenomenon across the country. As such, the improved match rate among physician addresses could represent the fact that the NP workforce is relatively new addition to integrated models of primary care that were until very recently physician practices. However, the lack of statistical significance in our sensitivity analysis contrasting address matches against the length of time since a provider last updated their information with CMS shows that the available data to test this theory are poor. Continued evaluation of these trends over time could confirm the potential suitability of the PCMH employment file as the de facto standard for measuring positional accuracy in workforce distribution estimates given that these data are publicly available. Lastly, our analysis was state centric. Although SC’s medical home distributions mirror the current “cottage industry” practice landscape throughout the country [33], and its geographical distribution of disease and disparity trends among racial/ethnic minorities resemble national trends [34–42], further evaluations are warranted to confirm if these trends are exhibited regionally as well as nationally.

## Conclusion

The federal government now formally requires all state Medicaid agencies to establish network and access standards for beneficiaries in effort to ensure adequate access to primary care and a host of other specialty providers and services. The use of external sources for validating provider practice locations has the potential to add real value to the updated federal rule given that MCOs suffer the sample problem of not requiring providers to update, in a timely manner, changes of employment information. Additionally, when hospitals purchase practices, generic hospital names often replace practice names within data systems, thereby exacerbating the mismatches. As both NPI and PCMH data files are routinely updated and freely available, these data could be used to regularly (e.g. annually) estimate the spatial accuracy of primary care provider distributions on a state-wide basis. At the same time, these data are limited for monitoring NP practice locations owing to the lack of distinction over place of practice. Unless there is widespread improvement by state agencies to begin recording where NPs practice, there is a strong potential for artificially introducing spatial bias into network adequacy evaluations and workforce projections.

## Abbreviations

CMS: Centers for Medicare and Medicaid Services
HPSA: Health Professional Shortage Areas
LLR: Labor, licensing, and regulation
MMC: Medicaid managed care
NCQA: National Committee for Quality Assurance
NP: Nurse practitioner
NPI: National Provider Identifier
NPPES: National Plan and Provider Enumeration System
PCMH: Patient Centered Medical Home
ZCTA: ZIP code tabulated area
